# Surgical innovation—hopefully kinder to patients

**DOI:** 10.1093/jhps/hnag017

**Published:** 2026-05-21

**Authors:** Richard E Field

**Affiliations:** City St George’s University of London

It is almost two centuries since John Rhea Barton reported the first surgical osteotomy of the proximal femur [1]. The procedure was undertaken on a patient with an ankylosed hip joint, in an era when joint ankylosis was a recognized sequelae of infection or trauma. It would be 20 years before the introduction of ether anaesthesia and the operation was completed in 7 min. A few weeks later, Barton manipulated the limb to prevent osseous healing and induce a pseudarthrosis. Barton’s osteotomy was undertaken to help the patient better move his lower limb rather than to better load the hip joint.

Over the next 120 years, tuberculous infection remained a common cause of hip pathology [2]. The introduction of X-rays helped the medical community to differentiate such cases from those with conditions such as developmental dysplasia, Legg-Calve-Perthes disease, slipped femoral capital epiphyses, and the progression of osteoarthritic joint degeneration. Armed with these insights, surgical minds turned to devising interventions to improve hip joint geometry and loading. In the paediatric world, the varus derotation osteotomy (VDRO) became the intervention of choice to stabilize subluxing and dislocated hips in the management of cerebral palsy cases and to improve femoral head containment in children with Legg-Calve-Perthes disease [3]. Also, in conjunction with pelvic osteotomy to better manage developmental dysplasia of the hip [4–6]. While for patients with degenerate joint disease, McMurray’s displacement osteotomy [7] became the intervention of choice, until the advent of joint replacement.

Correction of proximal femoral deformity involves multiple surgical challenges. In a small proportion of cases, the only requirement is to modify femoral neck version and a subtrochanteric osteotomy with plate or nail fixation will suffice [8, 9]. In cases requiring correction of femoral neck inclination and version, bony realignment may alter leg length, compromise abductor muscle function, damage the blood supply to the femoral head, and medialize the femoral shaft. Even if the former complications are avoided, medialization of the femoral shaft will create additional challenges should hip replacement be required in later life [10].

In this issue, we present a paper from Frédéric Laude and Camille Vorimore [11] describing strategies to address these challenges while minimizing soft tissue disturbance and leave the patient with a more cosmetically acceptable scar. While the authors do include post-operative radiographs of patients who have undergone ‘Laude’s osteotomies’, the paper is presented as a description of a new surgical technique rather than a report on clinical outcomes. The reader will note that Laude’s strategies to valgarise the femoral neck and to reduce femoral anteversion both involve the interposition of a small allograft wedge between the transected surfaces of the host bone. It will be interesting to see whether osteotomy healing is affected by this separation of the native bone surfaces. While it is exciting and refreshing to read about Laude’s innovative strategies, reports on the authors’ clinical results and the outcomes achieved by early adopters must be awaited so that the wider orthopaedic community can evaluate the clinical benefits and limitations of Laude’s elegant techniques.

On the topic of grafting osseous defects, we present a systematic review and meta-analysis from Dębiński and his colleagues [12] investigating the evidence for using synthetic bone grafts such as calcium sulphate, calcium phosphate, and hydroxyapatite; with or without adjuvants such as bone marrow concentrate, platelet rich plasma, or bone morphogenic protein in the treatment of osteonecrosis of the femoral head. While core decompression is a well-established intervention in the management of this condition, surgeon anxiety regarding the risk of femoral head collapse is understandable and the decision to fill the resulting bone void with a synthetic bone substitute is easy to rationalize. Perhaps counterintuitively, Dębiński’s analysis reveals that the there was no discernible difference in outcome when core decompression alone was compered to core decompression with synthetic bone graft or core decompression with synthetic bone graft and adjuvant. The authors did acknowledge that they were unable to drill down into specific graft and adjuvant combinations that may provide additional benefit to simple core decompression. Hopefully developments in orthobiologics and future studies will provide greater clarity on how to best manage this clinical condition. In the meantime, it will be interesting to see whether Dębiński‘s findings influence healthcare payers.

Members of our community who have been fortunate enough to attend lectures given by Reinhold Ganz will have marvelled at the boldness and elegance of the surgical interventions that he was able to conceive and execute. While Ganz often emphasized the imperative of preserving the blood supply to tissues, he was less eager to embrace what he viewed as a ‘peep show’ approach to surgery. One of the cited advantages of Laude’s proximal femoral osteotomy is that the procedure can be undertaken through the ‘bikini’ incision that is rapidly gaining popularity among direct anterior approach hip replacement surgeons. A similar strategy has been embraced by many surgeons undertaking peri-acetabular osteotomy. In this issue, we present a systematic review from McLeod and his colleagues [13] demonstrating that the conversion rate to total hip replacement is not adversely affected by adoption of minimally invasive approaches for peri-acetabular osteotomy. The authors also identified good improvements in patient reported outcomes but do not specify how these compared to the results after more extensile approaches. Once again, recognition was given to the benefit of less visible scarring, reflecting a trend of growing importance to Orthopaedic surgeons and patients, alike.

We hope that the papers in issue 13.2 of the *Journal of Hip Preservation Surgery* inspire and encourage you to investigate and develop hip preservation interventions to address and resolve hip pathology while minimizing physical and emotional disturbance to our patients.
